# DigestedProteinDB: A Compact and Scalable Key-Value Database for In Silico Peptide Digestion and Mass-Based Search

**DOI:** 10.1021/acs.jproteome.6c00152

**Published:** 2026-06-26

**Authors:** Toni Cvrljak, Janko Diminic, Jurica Zucko, Kresimir Krizanovic, Antonia Krsnik, Antonio Starcevic

**Affiliations:** † Faculty of Mining, Geology and Petroleum Engineering, University of Zagreb, 10000 Zagreb, Croatia; ‡ Faculty of Food Technology and Biotechnology, University of Zagreb, 10000 Zagreb, Croatia; § Faculty of Electrical Engineering and Computing, University of Zagreb, 10000 Zagreb, Croatia; ∥ School of Medicine, University of Zagreb, 10000 Zagreb, Croatia

**Keywords:** in silico digestion, mass spectrometry, peptide database, peptide mass fingerprinting, UniProt, RocksDB, protein identification, bioinformatics

## Abstract

Efficient comparison of experimental peptide masses with theoretical values is a central step in mass spectrometry (MS)–based proteomics, microbial biotyping, and MS imaging. Such analyses increasingly require fast and scalable access to large collections of in silico–digested peptides derived from large-scale and continuously evolving protein-sequence databases. Here, we present DigestedProteinDB, a compact and high-performance key–value database of peptides generated by enzymatic in silico digestion of UniProtKB/Swiss-Prot and TrEMBL sequences. Implemented using RocksDB, the system incorporates multiple optimization layers, such as peptide mass discretization and multistage storage compression, to minimize disk footprint and accelerate mass-range queries. In benchmark tests using 252 million UniProtKB protein sequences (5.9 billion peptides, trypsin; 6–50 aa; two missed cleavages), DigestedProteinDB required approximately 250 GB of disk space and operated within 16 GB of system RAM. Database construction required ∼2 days, and end-to-end mass-range queries (±0.1 Da) achieved a median latency of ∼7 ms per query across a batch of 10,000 randomly sampled queries. The resulting database can be used as a standalone local resource or integrated into bioinformatics pipelines for peptide mass fingerprinting (PMF), MS/MS-based protein identification, and microbial biotyping. Due to its modular design, new databases can be generated rapidly for different proteases, taxonomic subsets, or digestion parameters.

## Graphical abstract

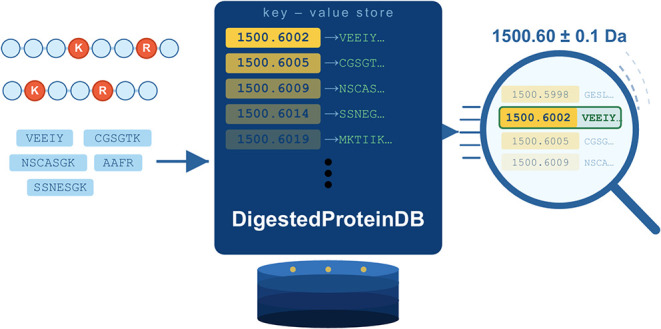

## Introduction

Mass spectrometry (MS) has become an indispensable technique in modern proteomics, microbial biotyping, and analysis of complex biological samples. A crucial step in interpreting mass spectra is the comparison of experimental peptide masses to theoretical values derived from known protein sequences, where databases play a central role.

One of the commonly used approaches, known as Peptide Mass Fingerprinting (PMF), employs algorithms that match measured peptide masses with theoretical spectra generated in silico from protein-sequence databases.^1^ Before identification in MS/MS analyses, proteins are typically cleaved into peptides by enzymes, such as trypsin or chymotrypsin. Publicly available protein-sequence databases such as UniProtKB/Swiss-Prot and TrEMBL^2^ have historically grown at a rapid pace, although recent releases have introduced redundancy reduction measures that have begun to stabilize or even reduce the size of TrEMBL. Regardless of the direction of these trends, efficient and rapid access to databases containing in silico-digested peptides remains a fundamental requirement for modern mass-spectrometry-based proteomics.

Traditional relational databases, while reliable and widely used in business and transactional systems, are not ideally suited for scientific workflows, in which a database is generated once and queried repeatedly. Such systems are generally not optimized for frequent, high-intensity searches using a single key—such as peptide mass—which results in increased latency and higher resource consumption.

This paper introduces a new approach for constructing and querying a compact, fast, and specialized database of digested peptides based on UniProt data sets. The proposed database, DigestedProteinDB, is implemented using RocksDB^3^ technology and optimized for high-speed searches by peptide mass.^3^ It incorporates multiple layers of data serialization and compression optimization, achieving an exceptional compactness and query efficiency.

The database is developed in the Java programming language and can be used either as a standalone Java library or as an independent Web application with REST and HTML interfaces, enabling remote access and easy integration into existing analytical systems.

The goal of this study is to describe the process of database construction, the optimization techniques applied, and the comparative results with existing solutions such as MaCPepDB,^4^ as well as to demonstrate the potential of DigestedProteinDB for applications in various forms of mass spectrometry-based analysis, including MS/MS identification and PMF.

### Overview of the MaCPepDB Database

MaCPepDB is a well-established peptide database designed to provide fast access to all tryptic peptides derived from the UniProtKB protein-sequence collection. The database performs enzymatic digestion of UniProtKB/Swiss-Prot and TrEMBL sequences in advance and enables peptide-centric searches, including mass-based queries, while associating peptides with protein accessions and taxonomic annotations. Its design is based on a relational database architecture implemented by using PostgreSQL, allowing the integration of rich metadata and advanced filtering capabilities.

The relational data model employed by MaCPepDB enables functionalities such as taxonomy-based filtering, peptide-property filtering, and incremental updates following new UniProtKB releases. In particular, MaCPepDB implements an update mechanism that avoids full database reconstruction by selectively processing newly added or modified protein sequences, which is advantageous for the long-term maintenance of a centralized and continuously evolving database resource.

However, this comprehensive and feature-rich design comes with substantial computational and storage requirements when applied on a proteome-wide scale. The construction and deployment of MaCPepDB were reported to require high-performance server hardware, including several multicore processors, hundreds of gigabytes of RAM, and multiple terabytes of SSD storage. As a result, MaCPepDB is primarily intended to operate as a centralized service rather than as a lightweight database that can be easily deployed locally or embedded into analytical pipelines.

DigestedProteinDB was developed to address a complementary use case. Instead of focusing on a single, comprehensive database covering all organisms and fixed digestion parameters, DigestedProteinDB is designed as a compact, modular, and locally deployable key–value database optimized for mass-based peptide searches. Its primary objective is to minimize disk footprint and memory usage while enabling fast access to in silico–digested peptides, making it suitable for use on standard laboratory hardware and for integration into bioinformatics workflows as a local resource.

A fundamental conceptual difference between the two approaches lies in their primary deployment models and architectural focus. MaCPepDB is explicitly designed and optimized to serve as a comprehensive centralized global resource. While its open-source codebase theoretically allows users to deploy it locally to modify parameters, like peptide length or the number of missed cleavages, before initiating the massive database construction, its heavy relational architecture is specifically tailored for maintaining a single, global tryptic digest on high-performance server infrastructure. In contrast, DigestedProteinDB is planned from the ground up to prioritize the rapid, modular generation of lightweight databases on standard laboratory hardware. Rather than relying on a monolithic global build, DigestedProteinDB allows researchers to swiftly generate experiment-specific databases for alternative proteolytic enzymes (e.g., trypsin, chymotrypsin, pepsin, or Glu-C), targeted taxonomic subsets, or customized digestion parameters. This modular approach encourages the creation of multiple agile databases tailored to particular sample types, entirely bypassing the computational bottleneck of rebuilding or hosting a proteome-wide data set. Another key difference is that MaCPepDB uses 64-bit integers to support 9 decimal places of precision, which contrasts with our 32-bit integer discretization to 4 decimal places. This difference highlights the complementarity of the two approaches, where MaCPepDB creators most likely prioritized high fidelity, while DigestedProteinDB prioritized compact storage and portable local deployment.

To provide some level of illustration in order to better understand the difference, the MaCPepDB database was reported to be constructed and hosted on a high-performance server equipped with 754 GB of RAM and multiple terabytes of SSD storage. The actual peak memory consumption of the database system during construction and querying was not explicitly reported. DigestedProteinDB was successfully constructed and queried on a system with 16 GB of RAM, including the operating system and the background processes.

To enable a meaningful baseline comparison, DigestedProteinDB was generated using the same digestion parameters as reported for MaCPepDB, including trypsin as the proteolytic enzyme, peptide lengths between 6 and 50 amino acids, and up to two missed cleavages. The slightly higher number of peptides observed in DigestedProteinDB reflects the growth of the UniProtKB database between the respective release versions used in the two studies rather than differences in digestion rules or filtering criteria (Table 1). In this dynamic data landscape, the ability to rapidly construct specialized databases tailored to specific experimental needs has become increasingly valuable for scientific workflows.

**Table 1:** Comparison of the Main Characteristics of the DigestedProteinDB and MaCPepDB Databases^a^

parameter	DigestedProteinDB	MaCPepDB
data set version	2025_04	2020_03
number of proteins	252,633,201	185,561,610
peptide length range	6–50 amino acids	6–50 amino acids
allowed missed cleavages	2	2
disk size	∼250 GB	Up to 8 TB
reported system RAM	16 GB	754 GB
average search time	<300 ms (mean)	<1 s
database build time	∼2 days	∼12 days

a DigestedProteinDB was constructed using the same digestion parameters as MaCPepDB (trypsin digestion, peptide lengths of 6–50 amino acids, up to two missed cleavages). Differences in the number of proteins and peptides reflect the use of different UniProtKB release versions rather than differences in database design.

In summary, MaCPepDB and DigestedProteinDB represent two complementary approaches to peptide database design. MaCPepDB emphasizes completeness, rich annotation, incremental updates, and centralized data management, making it well suited for large-scale, continuously maintained services. In contrast, DigestedProteinDB prioritizes compactness, modularity, and ease of local deployment, enabling rapid generation of experiment-specific peptide databases and flexible integration into mass spectrometry–based analytical pipelines. While a direct, quantitative performance benchmark between the two systems is challenging due to different UniProtKB versions and deployment modes, we can perform a meaningful comparison of their complementary architectural goals, which is summarized in the table below.

### Related Database and Search Approaches

Protein identification tools based on mass spectrometry, such as Mascot,^5^ X!!Tandem,^6^ MS-GF+,^7^ and Comet,^8^ typically operate directly on protein-sequence databases provided in formats such as FASTA. In these workflows, theoretical peptides or fragmentation spectra are generated on-the-fly and compared with experimental data. This strategy offers a high degree of flexibility and allows for straightforward customization of the search space for individual experiments.

However, on-the-fly digestion and indexing can become computationally demanding when repeatedly querying large proteome-wide databases containing tens of millions of protein sequences such as UniProtKB/TrEMBL. In such cases, database regeneration, indexing time, and resource consumption may represent practical bottlenecks, particularly in high-throughput or iterative analytical workflows.

DigestedProteinDB is positioned between fully dynamic sequence-based search tools and large, centralized peptide databases. It provides a precomputed peptide database that enables fast local mass-based searches while retaining the ability to regenerate databases efficiently when experimental parameters change. New databases can be constructed for alternative proteolytic enzymes, selected taxonomic groups (taxID), or modified digestion parameters such as peptide length or the number of allowed missed cleavages.

In this way, DigestedProteinDB combines key advantages of centralized resources, such as comprehensive peptide coverage and fast query performance, with the flexibility traditionally associated with on-the-fly database generation. The main contribution of this work is the development of a compact and efficient peptide database based on a key–value architecture that supports rapid mass-based searches, flexible database generation, and straightforward integration into existing bioinformatics pipelines.

The following sections describe the internal data structure of DigestedProteinDB, the database construction workflow, and the optimization strategies employed.

## Experimental Section

All source code was developed in the Java programming language and is publicly available in the repository: https://github.com/jankod/DigestedProteinDB.

### Database Data Structure

DigestedProteinDB is organized according to a key–value architecture, where the peptide mass is stored as the key, and the value contains a set of peptides sharing the same discretized mass.

Each value record includes the peptide sequence and a list of corresponding UniProt accession numbers of the source proteins. This structure enables rapid retrieval of all peptides whose masses fall within a specified range, along with their originating proteins—forming the foundation for subsequent protein identification in mass spectrometric analyses Figure 1.

**Figure 1:**
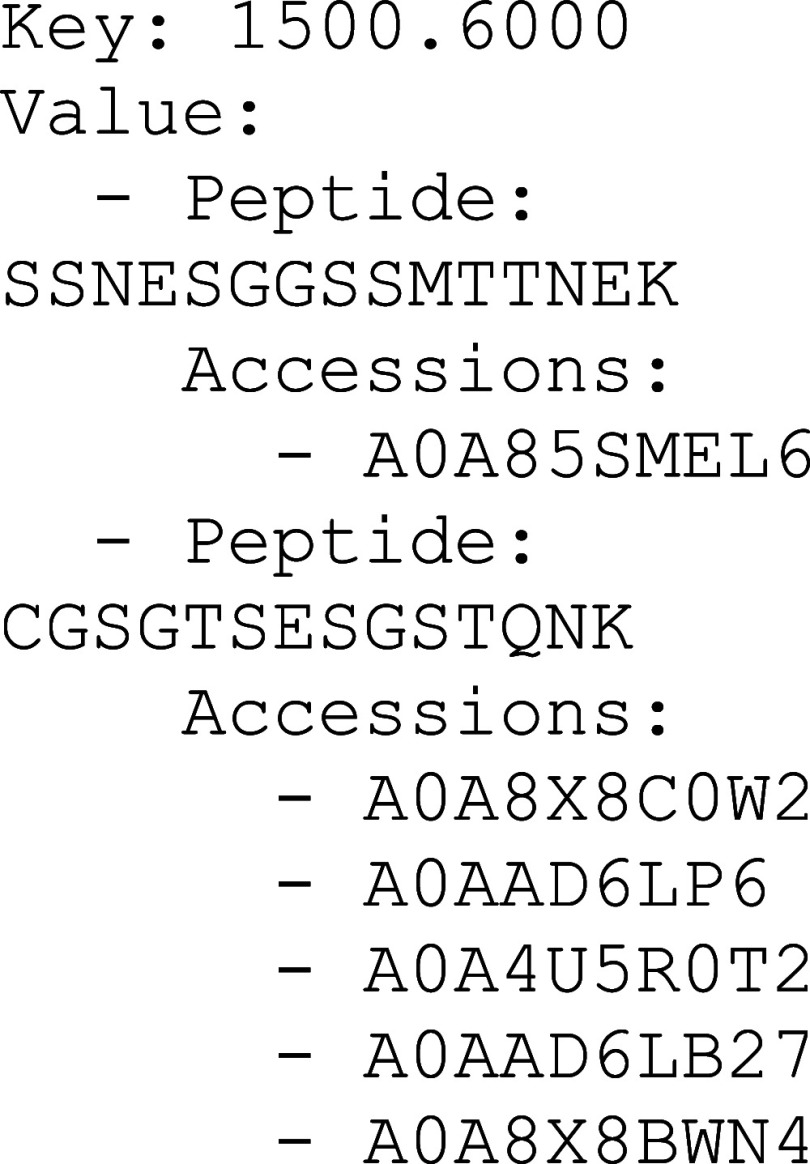
Hierarchical representation of the record structure used in DigestedProteinDB. Each discretized peptide mass serves as a key and is associated with one or more peptide sequences and their corresponding UniProt accession numbers stored as values.

In addition to the main key–value structure stored in the RocksDB system, a portion of auxiliary indices is maintained in memory using compact array-based data structures implemented in Java. These arrays provide mappings between internal numeric identifiers and encoded accession numbers or other metadata, enabling extremely fast retrieval of associated information without additional disk reads. This approach effectively combines the advantages of persistent on-disk storage with high-speed in-memory access.

### Database Construction

To build a new database, protein records from the UniProt database (Swiss-Prot and TrEMBL) were used. During the construction process, several fundamental parameters were defined:

- Organism set based on the NCBI taxonomy^9^ (a specific taxID or a higher taxonomic division, including all subordinate groups).
- Enzyme used for *in silico* protein cleavage (most commonly trypsin or chymotrypsin).
- Peptide length limits and the maximum number of missed cleavages were set according to the requirements of mass spectrometric analyses.

The database construction process proceeds through several intermediate stages designed to reduce the disk footprint and memory usage while improving search efficiency. To clarify the handling of incomplete digestion, all peptide sequences containing up to the maximum permitted number of missed cleavages (e.g., zero, one, or two) are explicitly and exhaustively generated during this initial in silico enzymatic cleavage step. These missed-cleavage variants are generated directly from the protein sequence and treated as independent primary entries; they are not inferred, concatenated, or aggregated during any subsequent postprocessing stages (as illustrated in step 1 of Figure 2). After this initial in silico cleavage, all explicitly generated peptides and their corresponding masses are stored in an intermediate CSV file.

**Figure 2:**
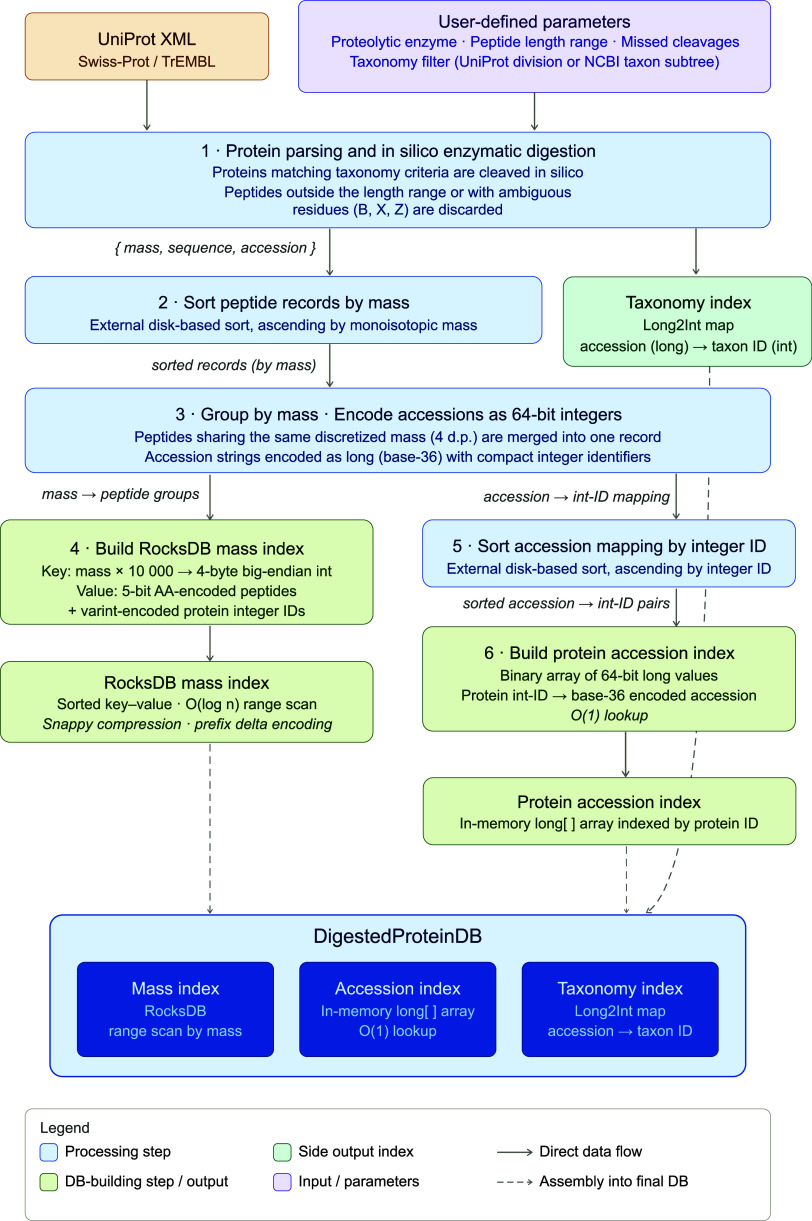
Overview of the DigestedProteinDB construction pipeline. Protein sequences from UniProtKB/Swiss-Prot or TrEMBL are filtered by an optional taxonomy criterion and digested *in silico* according to user-defined parameters (proteolytic enzyme, peptide length range, and allowed missed cleavages), yielding a stream of (mass, sequence, accession) records (Step 1). Records are externally sorted by monoisotopic mass (Step 2) and subsequently grouped by discretized mass (rounded to four decimal places), while accession strings are simultaneously encoded as 64-bit integers using a Base36 scheme and assigned compact internal integer identifiers (Step 3). The final RocksDB mass index is then built, using a 4-byte big-endian representation of mass × 10,000 as the key and a value composed of 5-bit amino acid–encoded peptide sequences and varint-encoded protein identifiers (Step 4). In parallel, the accession mapping is externally sorted by internal integer identifier (Step 5) and stored as an in-memory long­[] array that supports O(1) resolution from internal identifier to the original UniProt accession (Step 6). A taxonomy index mapping accession to NCBI taxonomy identifier is produced as a side output during parsing and is retained only when taxonomy-filtered queries are required. The final system consists of the on-disk RocksDB mass index combined with the in-memory accession index; the taxonomy index is optional.

During processing, the data are repeatedly sorted and grouped by peptide mass, while a series of optimization steps are applied—including rounding masses to four decimal places, removing duplicates, and serializing arrays of peptides associated with the same mass.

This multiphase approach (iterative loading, sorting, and rewriting of data) enables efficient memory management and progressive reduction of file size before the final storage in the RocksDB database. Such a design minimizes the need for a large memory capacity during database creation, making the process suitable even for systems with limited computational resources.

An overview of the complete construction pipeline, including its principal inputs, processing steps, and output indices, is shown in Figure 2. The pipeline is organized into six sequential stages, deliberately separating the computationally expensive digestion and sorting phases (Steps 1–2) from the encoding and index-building phases (Steps 3–6). This separation allows individual stages to be restarted or reconfigured independently without requiring full regeneration of the preceding intermediate data, which is particularly useful during development or when adjusting encoding parameters.

### Techniques for Optimizing Database Compactness

To reduce disk footprint and maintain efficient query performance, several data reduction and storage optimization strategies were applied prior to final insertion into the RocksDB key–value store.

RocksDB was configured to use Snappy compression, which provides a practical balance between compression ratio and decompression speed. This configuration reduces on-disk storage requirements while minimizing decompression overhead during mass-range queries. In addition, RocksDB’s built-in prefix compression and delta encoding mechanisms were utilized to reduce redundancy among adjacent mass keys, which often differ only in their least significant digits due to mass discretization.

At the application level, duplicate peptide entries were removed prior to storage, and peptides sharing identical discretized masses were aggregated into unified value records. This grouping strategy significantly reduces redundant key entries and improves the lookup locality.

Although not required for single-node deployments, the architecture allows optional partitioning of the database across multiple RocksDB instances, enabling parallel processing when handling very large data sets. This design provides scalability without altering the core of the data model.

Together, these configuration and structural choices enable the compact storage of billions of peptide entries while preserving efficient mass-based retrieval.

#### Data Sorting

RocksDB stores records in a key-sorted order, which is essential for efficient mass-based retrieval. New records are first inserted into an in-memory structure (memtable), where keys are maintained in a sorted order. Once flushed to disk, data are written as immutable sorted string table (SSTable) files that preserve this key ordering.

RocksDB is based on a log-structured merge tree (LSM-tree)^10^ architecture, in which multiple sorted segments are periodically merged through a process known as compaction. Compaction reconciles overlapping key ranges across SSTables and maintains an optimized layout for the read operations.

In the context of DigestedProteinDB, where the peptide mass serves as the primary key, this sorted storage model enables efficient point lookups and rapid sequential scans across defined mass intervals. Such range-based access patterns are central to mass spectrometry workflows, where experimental peptide masses are queried within a specified tolerance window.

By leveraging the inherent ordering guarantees of the LSM-tree architecture, DigestedProteinDB achieves efficient mass-range querying without requiring additional external indexing structures.

#### RocksDB Optimization

RocksDB configuration parameters were adjusted to improve the write stability and read efficiency during database construction and querying. These adjustments included customization of the write buffer (memtable) size, enabling memory-mapped (mmap) file access, configuration of the block cache for frequently accessed data blocks, and use of Snappy compression for data blocks.

The write buffer configuration supports efficient ingestion of large volumes of peptide entries during database generation, while block caching and memory-mapped access improve the read performance for repeated mass-range queries. Snappy compression provides a balance between the compression ratio and decompression overhead, reducing disk usage without introducing substantial computational latency.

Together, these configuration choices support stable query latency and efficient resource utilization when handling data sets containing billions of peptide entries.

#### Discretization of Peptide Masses

In mass spectrometry–based proteomics, peptide mass matching is typically performed within a defined tolerance window, expressed either in parts per million (ppm) for high-resolution instruments or in sub-Da ranges for lower-resolution systems. To enable efficient grouping of peptides by mass while preserving practical search precision, peptide masses were discretized to four decimal places.

All peptide masses were calculated as monoisotopic masses and rounded to four decimal places. This level of precision is tailored to and matches the high-resolution data provided by modern instruments (e.g., Orbitrap) for current mass-based matching workflows. As a result, peptides falling within narrow mass intervals are grouped under identical discretized mass values, improving key density, and reducing index fragmentation.

Internally, discretized masses were represented using 32-bit IEEE-754 floating-point values. For database storage, the binary representation of these floating-point values was reinterpreted as 32-bit integers and used as keys within RocksDB. This approach preserves exact bitwise ordering while enabling efficient lexicographic comparison of keys within the key–value store.

Using integer-based key representation ensures deterministic ordering and avoids potential ambiguities associated with floating-point comparison semantics. This design is particularly beneficial in LSM-tree–based storage engines such as RocksDB, where key comparison operations are performed frequently during both lookup and compaction phases.

#### Peptide Serialization

In conventional character-based representations, peptide sequences are stored using one 8-bit character per amino acid symbol. Because the 20 standard amino acids can be uniquely represented using only 5 bits, each residue was encoded using a compact 5-bit scheme, resulting in an approximate 37.5% reduction in raw sequence storage compared to byte-based character encoding.

Encoding was performed sequentially by packing residues into a contiguous binary bit stream stored within a byte array. This approach eliminates the overhead associated with Java string objects and reduces memory fragmentation by avoiding per-character object allocation.

The resulting binary representation allows efficient storage and direct decoding of peptide sequences during query processing while maintaining compatibility with the underlying key–value storage model.

#### Varint Serialization

When serializing numeric values such as peptide counts, sequence lengths, or internal identifiers, a fixed 32-bit integer representation can introduce unnecessary overhead, as most of these values are typically small. To reduce the storage size, variable-length integer (varint) encoding was employed.

In variant encoding, smaller integers are stored using fewer bytes, while larger values expand to multiple bytes as needed. This approach significantly reduces storage requirements when the majority of serialized values fall within low numeric ranges, as is common for peptide-associated metadata (e.g., short sequence lengths or limited accession counts per mass).

Varint encoding is widely used in compact binary serialization formats, including Google Protocol Buffers, because of its balance between space efficiency and straightforward decoding.

#### Accession Number Serialization

UniProt accession numbers consist of alphanumeric characters (A–Z, 0–9), corresponding to 36 possible symbols. To enable compact storage, accession strings were encoded using a Base36 scheme and represented as 64-bit integer values (long). This encoding preserves the full information content of the original accession while reducing storage overhead compared with character-based representations.

Within the main key–value store, each accession was assigned a 32-bit internal identifier (int). These internal identifiers were used in peptide value records to minimize storage size. A separate lookup structure was maintained in memory using a long­[] array, where the array index corresponds to the internal identifier and the stored value represents the encoded accession number.

This two-level mapping strategy significantly reduces memory usage within the key–value store while enabling constant-time resolution of internal identifiers to their original accession values. By avoiding storage of Java String objects for each peptide–accession association, the approach minimizes object overhead and improves memory locality.

## Web Application and API Access

To facilitate evaluation and integration of DigestedProteinDB, a web-based interface and REST API were developed and are publicly accessible at https://digestedproteindb.pbf.hr.

The web interface allows users to perform mass-based searches using configurable parameters including enzyme selection, taxonomic scope, peptide length limits, and the number of allowed missed cleavages. Preconstructed database instances corresponding to common parameter sets are also available for download.

In addition to the graphical interface, a REST API provides programmatic access to the database. Queries are submitted using HTTP GET requests with defined search parameters, and results are returned in the JSON format. This design enables straightforward integration into bioinformatics workflows and automated analysis pipelines.

To facilitate integration into custom analytical pipelines, a reference Python client script demonstrating programmatic access to the REST API is provided in the project GitHub repository (python directory). The example retrieves matched peptides and their associated UniProt accessions for a given precursor mass and tolerance window (https://github.com/jankod/DigestedProteinDB/tree/master/python).

## Results

The publicly available DigestedProteinDB instance hosted at https://digestedproteindb.pbf.hr was generated from UniProtKB/TrEMBL protein records (release 2025_04) using trypsin digestion with peptide lengths between 6 and 50 amino acids and up to two missed cleavages. A total of 252 633 201 protein sequences were processed, yielding approximately 5.9 billion peptide entries. After application of discretization, deduplication, serialization, and compression strategies, the resulting RocksDB database occupies approximately 250 GB of disk space. The database is fully operational on systems equipped with 16 GB of RAM, including the operating system and the background services.

The applied serialization strategies contributed substantially to database compactness. To precisely quantify the individual contribution of each encoding layer, an ablation study was performed on the Swiss-Prot subset, in which each encoding strategy was independently disabled and the resulting database size was compared against the fully encoded baseline (Table 2). The baseline configuration disables RocksDB block compression because Snappy and ZSTD compression were observed to slightly increase the on-disk size at the Swiss-Prot scale due to the high entropy of the application-level encoded data. Among the individual encoding layers, the 5-bit amino acid representation contributed the largest share of the total size reduction (24.3%), followed by varint serialization of numeric fields (11.7%) and the Base36 + two-level integer accession mapping (9%). The Base36 configuration additionally maintains a compact in-memory long­[] accession index (∼4.4 MB for Swiss-Prot, 572,348 entries), trading a modest amount of disk space for reduced memory overhead during query resolution.

**Table 2:** Ablation Study of Individual Encoding Contributions to Database Size, Evaluated on the Swiss-Prot Subset^a^

configuration	size (MB)	Δ vs baseline (MB)	Δ vs baseline (%)
full encoding (baseline)	736	-	-
no 5-bit peptide encoding	915	+179	+24.3%
no varint serialization	822	+86	+11.7%
no Base36 + integer index mapping^b^	802	+66	+9%

a The no-compression baseline (no RocksDB block compression) serves as the reference configuration, as Snappy and ZSTD compression increased database size at this scale due to the high entropy of application-level encoded data.

b No Base36 configuration eliminates the in-memory long­[] accession index (572,348 entries, ∼4.4 MB RAM for Swiss-Prot), trading disk efficiency for reduced memory overhead.

### Query Performance Benchmark

To precisely characterize end-to-end query performance, a batch benchmark of 10,000 mass-range queries was executed against the complete UniProtKB/TrEMBL trypsin database. Query masses were sampled uniformly from the full database range (360.1393–8,532.7586 Da), and each query was issued with a tolerance window of ±0.1 Da. Prior to measurement, 1000 warm-up queries were performed to allow the JVM (Java Virtual Machine) just-in-time compilation to stabilize.

Each measured query represents the full end-to-end operation, including (i) the RocksDB key–value range scan, (ii) deserialization and decoding of all returned peptide records (5-bit amino acid decoding and varint parsing), and (iii) resolution of internal integer identifiers to their corresponding UniProt accession strings. Queries were executed sequentially in a single thread; no parallelism was applied.

The benchmark was executed on a workstation equipped with an AMD Ryzen 9 9950X CPU, 32 GB of system RAM, and a Samsung 990 PRO 2 TB NVMe M.2 SSD. The RocksDB block cache was configured to 8 GB, and the JVM heap was fixed at 16 GB (-Xmx16g -Xms16g) to ensure reproducibility across runs. The complete benchmark source code is available in the project repository (BenchmarkRunner.java).

Summary latency statistics are presented in Table 3. The full per-query distribution is shown in Figure 3.

**Figure 3:**
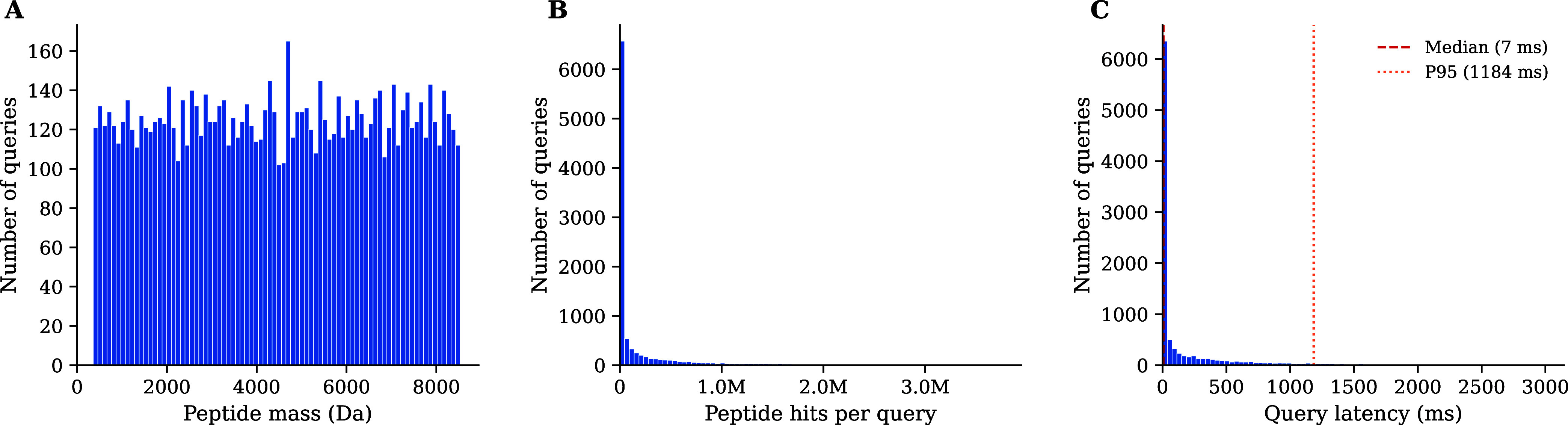
Benchmark characteristics of 10,000 mass-range queries (±0.1 Da) against the complete UniProtKB/TrEMBL trypsin database. (A) Uniform distribution of query masses across the full database range (360–8533 Da). (B) Distribution of peptide hit counts per query, exhibiting heavy-tailed behavior driven by nonuniform peptide density across the mass range. (C) Query latency distribution, mirroring the hit-count distribution and confirming that latency is governed by result set size. Vertical lines indicate the median (7 ms) and 95th percentile (1,184 ms).

**Table 3:** Query Latency Statistics for a Benchmark of 10,000 Randomly Sampled Mass-Range Queries against the Complete UniProtKB/TrEMBL Trypsin Database^a^

metric	value
total (10k queries)	2,004.27 s
mean per query	200.42 ms
median (P50)	6.68 ms
P95	1,184.34 ms
P99	2,045.30 ms
Max	2,995.89 ms

a Query masses were drawn from the range 360.1393–8,532.7586 Da with a mass window of ±0.1 Da. Prior to measurement, 1000 warm-up queries were executed to allow JVM just-in-time compilation to stabilize.

The latency distribution is highly skewed (Figure 3C), reflecting the nonuniform distribution of peptide entries across the queried mass range. Queries targeting mass intervals with low peptide density return results within a few milliseconds, whereas queries falling within the densely populated regions characteristic of tryptic digests (approximately 800–2500 Da) retrieve and deserialize substantially larger result sets, producing elevated latencies at the upper percentiles. This relationship is evident from the close correspondence between the per-query hit-count distribution (Figure 3B) and the latency distribution (Figure 3C). Consequently, the median query time (6.68 ms) provides the most representative estimate of typical end-to-end query performance, whereas the P95 and P99 values characterize worst-case behavior at the densest mass regions of the database.

The TrEMBL–trypsin database can be accessed through the web interface, queried programmatically via the REST API, or generated locally using the provided Java library.

## Discussion

The results demonstrate that a compact key–value architecture can support efficient mass-based querying at proteome-wide scale while remaining deployable on standard hardware. By precomputing peptide entries and organizing them according to discretized mass values, DigestedProteinDB enables rapid retrieval of candidate peptides within defined mass intervals, which is central to MS and MS/MS identification workflows. The benchmark results confirm that typical end-to-end query latency remains in the millisecond range (median: 6.68 ms) for a 5.9-billion-peptide database, while worst-case latencies are bounded by the size of the returned result set rather than by system overhead.

Unlike general-purpose relational database systems, which are designed to support complex transactional workloads and dynamic updates, DigestedProteinDB is optimized for read-intensive access patterns. In typical mass spectrometry workflows, peptide databases are constructed once and queried repeatedly, without modification. The absence of transactional overhead and the use of a simplified data model align the database architecture with the usage pattern.

### Advantages and Limitations

A principal advantage of the proposed approach is the combination of compact storage and efficient range-based query. The applied serialization and encoding strategies allow the storage of billions of peptide entries within a disk footprint manageable on standard workstations. This makes the system accessible to laboratories without a high-performance server infrastructure.

However, the simplified key–value model introduces inherent structural limitations. In contrast to relational database systems, DigestedProteinDB does not support dynamic schema modification, such as the addition of new columns or attributes after database construction. Similarly, the creation of secondary indices for arbitrary metadata fields is not natively supported within the core storage layer. As a result, complex multiattribute queries must be implemented externally or addressed through the generation of specialized database instances.

Furthermore, the database is not designed for incremental updates in the manner of a centralized relational system. When input data sets, digestion parameters, or structural attributes change, full database regeneration is required. While this approach simplifies the internal design and ensures structural consistency, it may be less suitable for continuously evolving centralized services that require frequent schema modification or real-time updates.

These trade-offs reflect a deliberate design decision to prioritize read-intensive performance and compactness over transactional flexibility and schema extensibility.

A characteristic property of the system is the heavy-tailed distribution of query latencies observed in the benchmark. Because the discretized mass keys in RocksDB are arranged in strictly sorted order, the lookup cost of locating the lower bound of a mass window is effectively constant; the dominant contribution to latency is therefore the deserialization and accession-resolution cost, which scales linearly with the number of peptides returned. As a consequence, queries targeting sparsely populated mass regions complete in only a few milliseconds, whereas queries within the densely populated tryptic mass band (approximately 800–2500 Da) can return substantially larger result sets and thus exhibit elevated latencies. This behavior should be regarded as a structural property of the underlying peptide distribution rather than a limitation of the storage engine itself.

### Intended Use and Deployment Model

Although a complete UniProtKB-based instance is publicly available for demonstration and benchmarking purposes, DigestedProteinDB is not primarily intended to function as a centralized online resource. Its main purpose is to enable the rapid generation of experiment-specific peptide databases that can be deployed locally and integrated directly into search tools or analytical pipelines.

In this deployment model, researchers or software developers generate database instances based on selected enzymes, taxonomic scopes, and digestion parameters relevant to their experimental design. This strategy reduces the unnecessary search space, improves computational efficiency, and aligns the database structure with the biological context of the analysis.

Regarding our proposed deployment model, a critical consideration is the handling of post-translational modifications (PTMs). Standard sample preparation protocols routinely introduce modifications, such as the carbamidomethylation of cysteine residues and the variable oxidation of methionine, which alter the expected masses of the resulting peptides. Explicitly generating and storing all combinatorially possible modified peptide sequences would inevitably lead to an exponential explosion of data volume. Accommodating this vast, dynamic search space natively within the core index would fundamentally conflict with the primary objective of DigestedProteinDB, which is to provide a compact, highly scalable, and locally deployable system capable of running on standard laboratory hardware without the need for a high-performance server infrastructure. Instead of centralized enumeration, DigestedProteinDB indirectly addresses PTMs through a deliberate design choice that favors modularity and pipeline integration. Users and developers can account for modifications using two primary strategies. First, for static or globally expected modifications (e.g., fixed carbamidomethylation), researchers can construct an experiment-specific database instance by supplying a custom or preprocessed protein-sequence FASTA file prior to the in silico digestion step. Second, for variable modifications (e.g., methionine oxidation), the extremely low latency of the key-value mass-range query allows downstream search algorithms to dynamically account for PTMs at the lookup stage. By querying the theoretical mass offsets (the mass “extras” corresponding to specific PTMs) alongside the unadjusted precursor mass, search engines can efficiently filter candidates. This separation of concerns ensures that the core database remains exceptionally fast and lightweight, leaving the complex, heuristic accounting of variable PTMs to the downstream matching and scoring algorithms, where they are most effectively managed.

### Applications and Future Perspectives

DigestedProteinDB is applicable to a range of mass spectrometry–based workflows, including peptide mass fingerprinting, MS/MS-based protein identification, microbial biotyping, and mass spectrometry imaging. In these contexts, rapid retrieval of candidate peptides within defined mass windows is essential for efficient downstream scoring and identification.

Future extensions may include support for additional enzymes, alternative mass calculation schemes, integration of selected metadata fields, or incorporation into hybrid workflows that combine precomputed peptide databases with dynamic spectral search algorithms.

## Conclusions

In this study, we introduced DigestedProteinDB, a compact and modular peptide database generated through in silico enzymatic digestion of large protein-sequence collections. By combining a key–value storage architecture with targeted serialization and encoding strategies, the system enables efficient mass-based peptide retrieval while maintaining a manageable disk footprint and memory usage.

Unlike centralized relational resources, DigestedProteinDB is designed to facilitate the rapid generation of experiment-specific databases tailored to defined enzymes, taxonomic scopes, and digestion parameters. This deployment model supports flexible integration into mass spectrometry–based analytical workflows, including peptide mass fingerprinting and MS/MS-based protein identification.

The publicly available UniProtKB-scale instance demonstrates that proteome-wide peptide databases can be constructed and queried on standard hardware without requiring high-performance server infrastructure. The principal contribution of this work lies in providing a portable and adaptable database architecture aligned with the read-intensive nature of mass spectrometric peptide search tasks.

Future development will focus on extending support for additional enzymatic rules, alternative mass calculation schemes, and deeper integration with MS/MS analysis tools and bioinformatics pipelines.

## Data Availability

The complete source code for DigestedProteinDB, including build instructions and example configurations, is publicly available at https://github.com/jankod/DigestedProteinDB. Prebuilt database instances and a publicly accessible demonstration server are available at https://digestedproteindb.pbf.hr. The UniProtKB release versions used for database construction are specified in the manuscript. All database instances can be regenerated locally using the provided source code and documented build procedure.
